# Paternal Engagement in Infant and Young Child Feeding: A Systematic Review and Meta‐Analysis of Its Extent and Associated Factors

**DOI:** 10.1111/mcn.70174

**Published:** 2026-02-25

**Authors:** Molalign Aligaz Adisu, Bogale Molla, Abraham Dessie Gessesse, Dagnew Tigabu, Tilahun Wodaynew, Abubeker Seid Ali, Tesfaye Engdaw Habtie

**Affiliations:** ^1^ Department of Pediatrics and Child Health Nursing, College of Health Sciences Woldia University Woldia Amhara Ethiopia; ^2^ Department of Nursing, College of Health Sciences Woldia University Woldia Amhara Ethiopia

**Keywords:** associated factors, Ethiopia, infant and young child feeding, meta‐analysis, paternal engagement

## Abstract

Optimal infant and young child feeding (IYCF) is a critical intervention during the first 1000 days of life, yet in Ethiopia, caregiving roles remain heavily gendered, often excluding fathers. This systematic review and meta‐analysis quantified the extent of paternal engagement in IYCF across Ethiopia and identified key associated factors. Following PRISMA guidelines, a comprehensive search of major databases and gray literatures identified 11 cross‐sectional studies involving 6030 fathers. Using a random‐effects model, the pooled prevalence of paternal engagement was 41% (95% CI: 25%–57%). Significant predictors of increased engagement included having an education above secondary level (AOR 4.27), smaller family size (AOR 4.03), first‐born child (AOR 3.36), and positive perceptions toward IYCF (AOR 2.68). Other factors included positive cultural beliefs (AOR 2.35), good IYCF knowledge (AOR 2.21), and the child being male (AOR 2.09). This study calls for shifting Ethiopia's nutrition strategies from mother‐centric to family‐centric models by implementing father‐inclusive, culturally sensitive programs. Training health extension workers to challenge gender norms through counseling and community dialog will address socio‐cultural barriers, promote shared caregiving, and improve child feeding outcomes.

## Summary

- Paternal engagement in infant and young child feeding in Ethiopia is low, with a pooled prevalence of 41%.
- Higher paternal education, good IYCF knowledge, and positive perceptions toward feeding practices are the strongest predictors of engagement.
- Entrenched patriarchal norms and a preference for male children significantly limit father involvement.
- National nutrition strategies should shift from mother‐centric to family‐centric models by training health extension workers to deliver father‐inclusive education and address restrictive gender norms.

## 1 Introduction

The first 1000 days of a child's life from conception until the age of 2 years is a critical window of opportunity for nurturing the optimal growth, health, and brain development (Victora et al. 2016). During this period, nutrition interventions have the greatest impact on shaping the fate of a child, affecting not only their physical stature but also their education, economic productivity, and long‐term disease risk (Victora et al. 2021). Infant and young child feeding (IYCF) practices, as defined by the World Health Organization (WHO) and United Nations Children's Fund (UNICEF), are key interventions during this period. These practices include early initiation of breast feeding, exclusive breast feeding until the first 6 months, and timely introduction of adequate, safe, and nutrient‐dense complementary foods while continuing breast feeding up to 2 years or more (World Health Organization 2023; UNICEF 2021).

Despite global efforts, poor IYCF practices are still a serious public health issue, particularly in the low‐ and middle‐income countries (LMICs). Malnutrition in any of its forms under nutrition, micronutrient deficiencies, and overweight is accountable for nearly half of all mortality in under‐five children, and majority of those deaths occur in LMICs (GBD 2017 Diet Collaborators 2019). Poor IYCF is a primary underlying cause of malnutrition. In sub‐Saharan Africa, for instance, rates of exclusive breastfeeding and minimum acceptable diet continue to lag behind the world's goals, contributing to the highest burden of stunting, wasting, and death (UNICEF 2021). This global crisis emphasizes the need for evidence‐based, multi‐faceted strategies to improve IYCF practices.

Traditionally, child cares including feeding has been almost exclusively left to mothers. Global health programs and scholars have historically focused on maternal knowledge, attitudes, and practices, often overlooking the influential role of other family members (Moura and Philippe 2023; Wiradnyani et al. 2024). Contemporary understanding posits that fathers are not merely passive bystanders but are active participants with the capability to play a vital role in influencing maternal and child health outcomes. There are numerous ways paternal engagement can occur, ranging from emotional support and decision‐making to supplying resources for healthy foods and direct involvement in feeding activities. This engagement matters. Research works demonstrate that positive paternal involvement is associated with improved initiation and continuation of breastfeeding, improved child dietary diversity, and reduced risk of childhood under‐nutrition (Agrawal et al. 2022; Zhou et al. 2024; Yargawa and Leonardi‐Bee 2015). Fathers can influence feeding patterns by creating a supportive environment for the mother, sharing childcare responsibilities, and involved in health‐related decisions, thereby alleviating maternal stress and enabling better childcare practice (Berhane et al. 2023; Saaka et al. 2023). Excluding the father, therefore, is a missed opportunity for strengthening child nutrition interventions.

Ethiopia, as the second most populous African countries, has made significant strides in improving child health indicators in the past two decades. With the implementation of the Health Extension Program and other national nutrition interventions, child mortality and stunting have reduced (Central Statistical Agency CSA Ethiopia & ICF 2017). Despite this, the burden of malnutrition remains unacceptably high. According to the 2024–2025 Ethiopia Demographic and Health Survey (EDHS), 40% of under‐five children are stunted and 5% are wasted (Central Statistical Agency CSA Ethiopia & ICF 2026). These numbers reflect a critical public health concern.

Meanwhile, IYCF practices among Ethiopians are inadequate. While the initiation of breastfeeding at an early age is relatively high, exclusive breastfeeding during the first 6 months and the minimum acceptable diet are low, at 57% and 16.2%, respectively (Central Statistical Agency CSA Ethiopia & ICF 2026). This indicates a wide gap between the qualities of feeding in the most vulnerable period of child's life. The factors of such poor practices are multifaceted, among which are food insecurity, low levels of maternal education, parental perception to IYCF, and ingrained socio‐cultural norms (Geda et al. 2021; Khan et al. 2017; Tadesse et al. 2023; Tololu et al. 2025). In such a multi‐faceted scenario, the Ethiopian father is shaped by a unique interplay of tradition and modernity. Ethiopian society is predominantly patriarchal; with fathers having the primary authority in household decision‐making, including allocation of resources toward food and healthcare (Bilal et al. 2016). Understanding how this authority and influence are exercised in the realm of IYCF is paramount.

Within the Ethiopian context, the evidence base on paternal engagement in IYCF is characterized by two critical gaps: first, a lack of a consolidated national picture of the extent of engagement, as existing evidence is small‐scale, localized studies, and second, a fragmented and often contradictory understanding of its associated factors. This research directly addresses these gaps by conducting the first comprehensive systematic review and meta‐analysis to establish a definitive quantitative baseline of engagement levels and to identify the most powerful and consistent predictors of paternal involvement. The synthesized findings will provide concrete evidence to strategically inform national nutrition policies and father‐inclusive programming for stakeholders like the Federal Ministry of Health, guide the development of targeted interventions for health extension workers, and ultimately serve as a catalyst for a fundamental paradigm shift in child nutrition strategies–moving from a traditionally mother‐centric model to a holistic, family‐centric approach that formally recognizes and empowers fathers as vital agents of IYCF.

## 2 Methods

### 2.1 Study Design and Reporting

This systematic review and meta‐analysis were conducted to estimate the pooled level paternal engagement and its associated factors in IYCF in Ethiopia. The review was reported following the Preferred Reporting Items for Systematic Reviews and Meta‐Analyses (PRISMA) guidelines. The research protocol was registered prospectively with the International Prospective Register of Systematic Reviews (PROSPERO) under the registration number CRD420251164639 to prevent duplication of efforts and guarantee transparency.

### 2.2 Search Strategy and Sources of Information

A systematic and comprehensive search was built following the “PEOS” format (Population, Exposure, Outcome, Study design, and Setting). The search was conducted in major international electronic databases, including PubMed/MEDLINE, Scopus, Web of Science, Cochrane Library, and Embase. Gray literature was also searched through university repositories and organizational websites to enhance the comprehensiveness of the evidence base. The search strategy used a combination of Medical Subject Headings (MeSH) and free‐text keywords, connected by Boolean operators (AND, OR). The core search term was constructed as: (“father” OR “paternal” OR “male partner”) AND (“engage” OR “involve” OR “participate”) OR (“role”) AND (“infant and young child feeding” OR “IYCF” OR “complementary feeding” OR “breastfeeding”) AND (“Ethiopia”). This strategy was designed to comprehensively address the review's primary questions: “What is the extent of paternal engagement in IYCF in Ethiopia?” and “What are the associated factors of paternal engagement in IYCF?”

### 2.3 Inclusion and Exclusion Criteria

Research works were selected based on pre‐set eligibility criteria. We included all observational studies (case‐control, cross‐sectional, and cohort) that were carried out in Ethiopia up to September 2025 without language restriction. Fathers whose children were aged under‐5 years formed the study population. The primary outcome was the prevalence of fathers' engagement in IYCF, while secondary outcomes were predictors of fathers' engagement, expressed as adjusted odds ratios (AORs) with 95% confidence intervals (CIs). We did not include qualitative studies, editorials, conference abstracts, reviews, and studies whose full text was unavailable, low quality or where data could not be accessed for meta‐analysis.

### 2.4 Study Selection and Quality Assessment

The process of selecting the studies was independently performed by three reviewers (BM, DT, and ADG). All identified records were initially compiled, and duplicates were removed using EndNote version 25. The reviewers then screened titles and abstracts of the remaining articles for relevance. The full papers of likely eligible studies were subsequently retrieved and reviewed in full against the inclusion criteria. Any disagreement among reviewers was resolved by discussion and consensus with a third senior reviewer (MAA). Methodological quality and risk of bias of included studies were assessed using the Joanna Briggs Institute (JBI) adapted to cross‐sectional studies. The instrument assesses quality on representativeness of the sample, study subject selection, exposure and outcome measurement confounding factors, and statistical analysis. The studies that achieved greater than 70% were assigned a low risk of bias and were included in the final meta‐analysis.

### 2.5 Data Extraction and Statistical Analysis

A pre‐piloted standardized form for data extraction was employed to collect data from the included studies. Two independent reviewers (TW and ASA) extracted data like the first author's name, publication year, geographic region, design, sample size, prevalence of paternal involvement, and factors with their AORs and 95% CIs. Extracted data discrepancies were addressed through reference to the original article and consensus. The meta‐analysis was performed using STATA statistical software version 17. Estimation of the pooled paternal engagement level and pooled AORs were done using the random‐effects model, accounting for expected heterogeneity in socio‐cultural norms and measurement approaches. In this context, the pooled prevalence should be interpreted as a weighted average summary of paternal engagement across diverse populations rather than as a single uniform “national” value. Heterogeneity was statistically estimated using the *I*² statistic and Cochran's Q test, with a level of *I*² ≥ 50% assumed to indicate significant heterogeneity. Formal statistical tests for publication bias were not conducted, as funnel plots and Egger's regression are designed for comparative effect sizes and may be misleading for single proportions due to the bounded nature of prevalence estimates. To enhance the robustness of the pooled estimates, we conducted a comprehensive literature search including gray literature and performed sensitivity analyses.

## 3 Results

### 3.1 Literature Search

**Figure 1:**
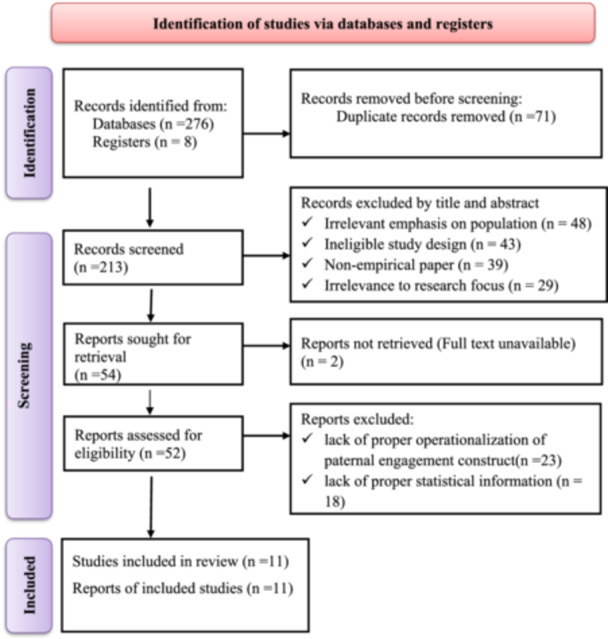
PRISMA flow diagram illustrating the study selection process for the systematic review and meta‐analysis on paternal engagement in infant and young child feeding in Ethiopia.

The systematic search was conducted as per PRISMA protocol across the core databases: PubMed/MEDLINE, Scopus, Web of Science, Embase, and the Cochrane Library. The title search yielded a total of 284 candidate articles. Upon exclusion of 71 duplicate records, 213 unique publications made it to title and abstract screening phase. In this screening phase 1, 159 records were excluded for a variety of reasons: irrelevant emphasis on population (*n* = 48), inappropriate study design mismatch (*n* = 43), non‐empirical paper type (*n* = 39), or outside scope of review content (*n* = 29). The remaining 54 articles made it to full‐text retrieval from which 52 articles were successfully downloaded. In‐depth analysis of these 52 articles in full text resulted in exclusion of 41 studies largely due to lack of proper statistical information for meta‐analysis (*n* = 18) or lack of proper operationalization of paternal engagement construct (*n* = 23). Finally, 11 studies met all inclusion and exclusion criteria and made it to final systematic review and meta‐analysis: PRISMA study flow diagram is shown in Figure 1.

### 3.2 Characteristics and Quality of the Included Studies

Eleven studies were included in this systematic review and meta‐analysis, all of which used a cross‐sectional study design (Abera et al. 2017; Bishaw et al. 2024; Bogale 2022; Bolka et al. 2025; Ganfure et al. 2025; Gemede 2025; Gobena 2018; Orkaido et al. 2025; Shimelis et al. 2024; Ethiopia Nigusu Tefera 2025; Wolkanto et al. 2023). Studies included in this review were published from 2016 to 2024, with most of them published in 2024. Overall, the studies assembled a large number of samples together with 6030 participants. The number of samples in each study demonstrates marked variation, ranging from the lowest sample size in Amhara and Southern Ethiopia region with 408 participants (Bogale 2022; Gobena 2018) to Oromia with 1152 participants (Shimelis et al. 2024), with a median sample size of 548 samples on average. Also, the number of samples geographically represents samples from various regions in Ethiopia, with a marked presence from the Southern Ethiopia, Sidama, Oromia, Amhara, and Central Ethiopia region. The level of Prevalence on Paternal Involvement in IYCF practice demonstrates wide disparities across studies, with the lowest prevalence reported at 26.2% in Oromia (Shimelis et al. 2024) and the highest at 72.4% in Southern Ethiopia (Abera et al. 2017), the highest level respectively. The methodological rigor of the included studies was assessed using the JBI critical appraisal checklist for analytical cross‐sectional studies. The overall quality was found to be high, as all 11 studies achieved JBI scores of 7 and 8 out of 8 (Table 1).

**Table 1:** Characteristics and quality assessment of studies included in the systematic review and meta‐analysis of paternal engagement in IYCF in Ethiopia.

Study (year conducted)	Region	Design	Sample size	Prevalence	JBI score
Wolkanto et al. (2023)	Southern Ethiopia	CS	593	50.90%	8
Bolka et al. (2025)	Sidama	CS	422	68.70%	8
Ganfure et al. (2025)	Central Ethiopia	CS	605	27.30%	8
Abera et al. (2016)	Southern Ethiopia	CS	417	72.40%	8
Gobena et al (2018)	Southern Ethiopia	CS	408	39.20%	8
Shimelis et al. (2024)	Oromia	CS	1152	26.20%	7
Ethiopia Nigusu Tefera (2025)	Oromia	CS	415	45.78%	8
Orkaido et al. (2025)	Southern Ethiopia	CS	415	52.20%	8
Bogale et al. (2022)	Amhara	CS	408	43.10%	8
Bishaw et al. (2024)	Amhara	CS	602	68.60%	8
Gemede et al. (2025)	Oromia	CS	593	54.10%	8

Abbreviation: CS, cross‐sectional.

### 3.3 Paternal Engagement in Infant and Young Child Feeding in Ethiopia

**Figure 2:**
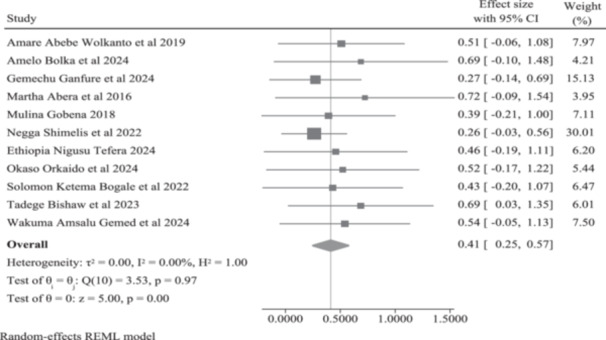
Forest plot showing the meta‐analysis of pooled prevalence estimates for paternal engagement in IYCF practices in Ethiopia.

**Figure 3:**
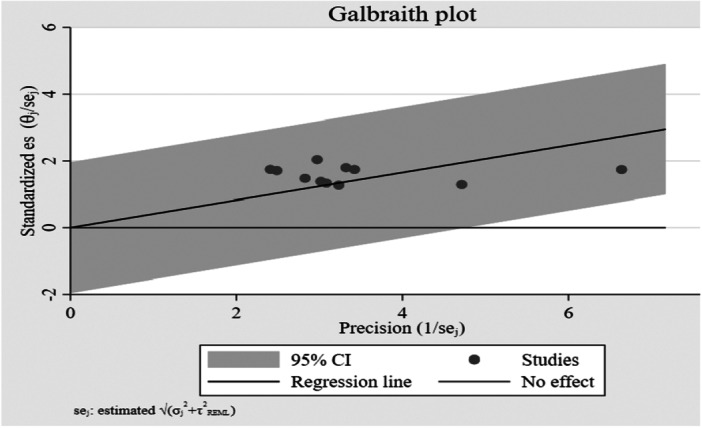
Galbraith plot for assessing heterogeneity in the meta‐analysis of paternal engagement in IYCF in Ethiopia.

The meta‐analysis of 11 studies using a random‐effects REML model yielded a pooled estimate for paternal engagement in IYCF in Ethiopia of 41% (95% CI: 25% to 57%), which was statistically significant (*z* = 5.00, *p* < 0.001). The included studies exhibited negligible heterogeneity (*I*² = 0.00%; Q (10) = 3.53, *p* = 0.97) (Figure 2). A Galbraith plot confirmed this homogeneity, with all study estimates fell within the 95% confidence band (Figure 3).

### 3.4 Sensitivity Analysis

**Figure 4:**
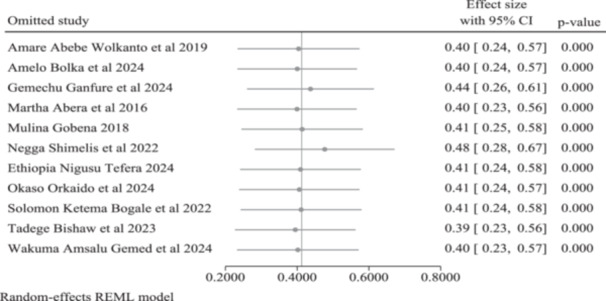
Leave‐one‐out sensitivity analysis of the pooled prevalence of paternal engagement in IYCF in Ethiopia.

A leave‐one‐out sensitivity analysis was conducted to assess the robustness of the pooled prevalence estimate of paternal engagement in IYCF. Sequential exclusion of individual studies did not materially alter the pooled estimate, which remained stable and statistically significant, ranging from 39% to 48%. These findings indicate that no single study exerted a disproportionate influence on the overall pooled prevalence (Figure 4).

### 3.5 Factors Affecting Paternal Engagement in Infant and Child Feeding in Ethiopia

Socio‐demographic and cultural factors were all significant predictors of a father's engagement in IYCF in Ethiopia. Specifically, fathers with positive cultural beliefs towards IYCF had significantly higher engagement (AOR = 2.35, 95% CI: 1.06–3.64, *p* < 0.001), and no heterogeneity was observed between the studies (*I*² = 0.00%). Birth order was a strong predictor, with fathers more than three times as likely to be involved with first‐born children as with subsequent births (AOR = 3.36, 95% CI: 1.48– 5.23, *p* < 0.001), with low heterogeneity between the studies (*I* ^2^ = 5.05%). Similarly, family size was also an important factor such that fathers with smaller families had more than three times higher adjusted odds of involvement (AOR = 4.03, 95% CI: 1.70– 6.37, *p* < 0.001), a finding which had moderate heterogeneity (*I*² = 44.31%). A child's sex also played role, with fathers more likely to be involved if they had male compared to female (AOR = 2.09, 95% CI: 1.29–2.89, *p* < 0.001), with low heterogeneity across studies (*I*² = 22.13%).

Furthermore, a father's individual attributes, including his education, knowledge, and perceptions, were also strongly associated to IYCF engagement. Educational level was a particularly strong predictor; fathers with more than secondary and above education had considerably more involvement (AOR = 4.27, 95% CI: 2.99–5.55, *p* < 0.001), and no heterogeneity between studies was detected (*I*² = 0.00%). Fathers with good IYCF knowledge also showed significantly higher feeding practice involvement (AOR = 2.21, 95% CI: 1.55–2.86, *p* < 0.001), with moderate heterogeneity observed between studies (*I*² = 40.50%). Finally, fathers with positive perceptions about IYCF had significantly higher levels of involvement (AOR = 2.68, 95% CI: 1.80–3.57, *p* < 0.001), as the analysis showed moderate heterogeneity across the included studies (*I*² = 52.33%) (Table 2). Detailed analyses are presented in Supporting Information File 1.

**Table 2:** Pooled estimates of determinants affecting paternal engagement in IYCF in Ethiopia.

Associated factors	Pooled effect size & 95% CI	*p*‐value	Heterogeneity (I^2^)	Studies pooled
Positive cultural beliefs towards IYCF	2.35 (1.06 to 3.64)	< 0.001	0.00%	Bogale et al. (2022); Gemede et al. (2025)
First‐born children	3.36 (1.48 to 5.23)	< 0.001	5.05%	Bishaw et al. (2024); Bogale et al. (2022); Ganfure et al. (2025)
Less than five family size	4.03 (1.70 to 6.37)	< 0.001	40.50%	Gobena et al. (2018); Ganfure et al. (2025); Shimelis et al. (2024)
Male child	2.09 (1.29 to 2.89)	< 0.001	22.13%	Bishaw et al. (2024); Bogale et al. (2022); Ganfure et al. (2025)
Above secondary educational status	4.27 (2.99 to 5.55)	< 0.001	0.00%	Gobena et al. (2018); Abera et al. (2017); Bishaw et al. (2024); Bogale et al. (2022); Bolka et al. (2025); Ganfure et al. (2025); Gemede et al. (2025)
Good IYCF Knowledge	2.21 (1.55 to 2.86)	< 0.001	40.50%	Ethiopia Nigusu Tefera 2025; Bogale et al. (2022); Bolka et al. (2025); Orkaido et al. (2025); Shimelis et al. (2024); Gemede et al. (2025)
Positive perception towards IYCF	2.68 (1.80 to 3.57)	< 0.001	52.33%	Ethiopia Nigusu Tefera 2025; Abera et al. (2017); Bogale et al. (2022); Bolka et al. (2025); Orkaido et al. (2025); Gemede et al. (2025); Wolkanto et al. (2023)

## 4 Discussion

This systematic review and meta‐analysis provide the first comprehensive quantitative synthesis of the extent and associated factors of paternal engagement in IYCF in Ethiopia. The pooled prevalence of paternal engagement was found to be 41%, indicating while substantial proportion of fathers are engaged, a significant gap remains, with nearly six out of ten children not fully benefiting from paternal support in this critical domain. Consistent 42% paternal engagement level was reported in Tanzania and Australia (Seko and Mosha 2024; Mallan et al. 2014). However, this is much lower than studies conducted in Ghana and India where paternal involvement in child care were about 64% and 60% respectively (Saaka et al. 2023; Mithra et al. 2021). Relative homogeneity is surprising given the regional diversity of Ethiopia but might imply that broad national socio‐cultural norms of fatherhood and gender roles are a more unifying influence than local differences. This consistency also makes the case for a unified national intervention.

The pooled analyses revealed that fathers with positive cultural beliefs towards IYCF had significantly higher engagement level than counter parts. In most Ethiopian cultures, IYCF was traditionally viewed as the exclusive role of mothers, with the father playing the primary role of provider (Bilal et al. 2016). What our result suggests is that when community norms shift to view feeding as a shared responsibility, fathers are more than twice as likely to participate. This is in line with evidence in Ghana, where community‐level sensitization was more powerful than counseling at the individual level in changing paternal behavior (Ntim Babae et al. 2025).

Fathers with first‐born child and those from smaller families had three‐ and four‐ fold increase in engagement in IYCF, respectively, compared to those with subsequent births and larger family size. This is, may be due to the constraints on resources—both attentional and economical. Paternal involvement is heightened by the novelty of the parental role and the urgent need for a cohesive parental unit. However, as family size increases and subsequent children arrive, the family's finite resources specifically time, economical, and emotional energy become stretched. This resource dilution typically compels the father to revert to the established primary role of financial provider, leading to a measurable decline in direct, hands‐on involvement with younger and later‐born siblings as compared to the initial experience with the first child (Jensen et al. 2017).

The pooled factor analysis revealed that Ethiopian fathers are far more likely to be involved in IYCF when the child is male compared to female, an inequality with its origins in deeply held cultural values and gender roles that position males at the nexus of the family. Additionally, male children are traditionally considered by most Ethiopian societies as carriers of the family lineage and future providers, which makes fathers more interested in caregiving and feeding practices for males. Such gender discrimination reflects broader societal values that allocate greater resources, attention, and responsibility to male children, shaping paternal behavior and engagement. This result is supported by a finding from India and South Asia, wherein father engagement in child care including nutrition and favor to male children (Rajan and Morgan 2018; Liyana Pathirana et al. 2025). Addressing this gendered disparity in paternal engagement is central to improving equitable child nutrition outcomes and entails culturally sensitive interventions that recognize and work within these normative contexts.

The results reveal that educated fathers were four times higher engagement level in IYCF practice than non‐educated fathers. This finding is robustly supported by global evidence (Bhattacharyya et al. 2023; Mithra et al. 2021). Higher education is logically linked with increased exposure to modern health knowledge, improved critical thinking, and shedding of rigid traditional male and female gender roles. Educated fathers are better able to understand the scientific underpinning of the optimal IYCF practices and are more likely to share intellectual and practical work with their partner. This underscores the essential long‐term value of investing in education, particularly for young men and boys. In short, IYCF communications materials must be designed to suit literacy levels and employ extensive use of visual aids and interactive methods for low‐literacy users.

Fathers with good knowledge and positive perception were twice more likely to be engaged in IYCF. Global evidences support this finding (Atkinson et al. 2021; Kaloro et al. 2025; Sachdeva and Gupta 2022). This relationship is justified by the fact that domain‐specific practical knowledge and positive perceptions are fundamental prerequisites for behavioral change, empowering fathers with the necessary competence and motivation to participate in IYCF. Knowledge provides the critical “how‐to” understanding of feeding practices, while positive perceptions foster a sense of responsibility and self‐efficacy, enabling fathers to overcome traditional gender norms that may discourage their involvement. Consequently, these cognitive factors directly facilitate the translation of paternal capacity into tangible, engaged feeding behaviors.

The pooled AORs must be interpreted by considering the confounding factors adjusted for in the original studies. Maternal education, household wealth/income, father's occupation, place of residence (urban/rural), and child age were among the most commonly adjusted variables in research. These are appropriate adjustments for key socio‐economic and demographic confounders. However, a number of potentially important confounding or mediating variables were inconsistently accounted for. These include maternal employment status, the quality of the marital relationship, maternal attitudes toward father involvement, and the father's own childhood experiences of caregiving. The absence of adjustment for these factors, particularly maternal attitudes which may gatekeep father involvement, means the reported AORs might partially reflect the influence of these unmeasured variables.

This review has certain limitations. First, all included studies were cross‐sectional in nature, which precludes excluding causality inferences. Second, measurement of paternal engagement also varied across studies; however, the observed low statistical heterogeneity provides reassurance regarding consistency of results. Future research should prioritize longitudinal or mixed‐methods designs to establish temporal and causal relationships between identified factors and paternal engagement. Such studies could also explore father‐specific intervention models and their long‐term effects on child nutritional status.

## 5 Conclusion and Recommendation

Paternal engagement in IYCF in Ethiopia is suboptimal not ideal but modifiable. It is strongly influenced by a combination of structural factors (education, family size), embedded cultural practices (child sex preference, cultural beliefs), and modifiable cognitive factors (knowledge, perception). The findings have direct programmatic implications for Ethiopia's Health Extension Program and national nutrition strategies. Health extension workers should be trained to engage fathers through targeted counseling, couple‐based education, and community dialog sessions that challenge restrictive gender norms. Father‐inclusive messaging should be integrated into existing maternal and child health platforms, such as growth monitoring and nutrition demonstrations. At the policy level, revising national nutrition guidelines to explicitly include fathers as key actors in IYCF can foster a systemic shift from mother‐centric to family‐centric care.

## Author Contributions

M.A.A. conceived the study idea and led the study. B.M., D.T., and A.D.G. screened the literature. T.W. and A.S.A. extracted data from eligible studies and conducted a critical appraisal of the included studies. M.A.A. and T.E.H. accessed and verified the data, having full access to all study data. M.A.A. analyzed and interpreted the data with significant contributions from T.E.H., with input from all authors. M.A.A. drafted the initial manuscript, and all authors provided critical revision, approved final version, and agreed on final responsibility for the publication decision.

## Funding

The authors received no specific funding for this work.

## Conflicts of Interest

The authors declare no conflicts of interest.

## Supporting information

Factors affecting paternal engagement in Infant and child feeding in Ethiopia.

## Data Availability

Data will be available upon the reasonable request of the corresponding author (MAA).
