# Supplementary material for: Paternal Engagement in Infant and Young Child Feeding: A Systematic Review and Meta‐Analysis of Its Extent and Associated Factors
Source: Matern Child Nutr. 2026 Feb 25;22(1):e70174. doi: 10.1111/mcn.70174 (PMC12935065; doi:10.1111/mcn.70174)
Supplement: Supplementary file 1 — Factors affecting paternal engagement in Infant and child feeding in Ethiopia. [file MCN-22-e70174-s001.docx]

**Factors affecting paternal engagement in Infant and child feeding in Ethiopia**

1. **Positive cultural beliefs towards IYCF**
2. **First Birth order**

1. **Less than five Family sizes**

1. **Male child**

1. **Secondary and above Education status**

1. **Good IYCF Knowledge**

1. **Good perception to IYCF**
